# Supplementary material for: An Interprofessional E-Learning Resource to Prepare Students for Clinical Practice in the Operating Room—A Mixed Method Study from the Students’ Perspective
Source: Healthcare (Basel). 2021 Aug 11;9(8):1028. doi: 10.3390/healthcare9081028 (PMC8393395; doi:10.3390/healthcare9081028)
Supplement: Supplementary file 1 [file healthcare-09-01028-s001.zip › healthcare-1322800-supplementary.pdf]

Questionnaire: E-learning resource "Introduction to the OR"

By filling out this questionnaire I give my consent to participation in the study

Age: ……………………………   Sex: ◯ female ◯ male ◯ other

Have you been at an OR-ward before your clinical placement? ◯ yes ◯ no

Have you completed the e-learning resource ”Introduction to the OR”?

◯ yes ◯ no **If no, thank you for your participation.**

If yes, continue to fill out the questionnaire.

On which digital unit did you carry out the learning activity?

◯ Computer ◯ Mobile phone ◯ Other

Grade to what extent you think that the e-learning resource helped you prepare for clinical practice in the OR?

◯ Very little extent ◯ Little extent ◯ Some extent ◯ Large extent ◯ Very large extent

Grade to what extent you think that the different parts of the e-learning resource helped you prepare for clinical practice in the OR?

OR-design

◯ Very little extent ◯ Little extent ◯ Some extent ◯ Large extent ◯ Very large extent

Hygiene routine

◯ Very little extent ◯ Little extent ◯ Some extent ◯ Large extent ◯ Very large extent

Professions working at the OR

◯ Very little extent ◯ Little extent ◯ Some extent ◯ Large extent ◯ Very large extent

Radiation safety

◯ Very little extent ◯ Little extent ◯ Some extent ◯ Large extent ◯ Very large extent

Gloving technique

◯ Very little extent ◯ Little extent ◯ Some extent ◯ Large extent ◯ Very large extent

Surgical hand preparation

◯ Very little extent ◯ Little extent ◯ Some extent ◯ Large extent ◯ Very large extent

Gowning procedure

◯ Very little extent ◯ Little extent ◯ Some extent ◯ Large extent ◯ Very large extent

Questionnaire: E-learning resource "Introduction to the OR"

By filling out this questionnaire I give my consent to participation in the study

To what extent did the e-learning resource "Introduction to the OR" contain the information you needed to be well prepared for your clinical placement?

◯ Very little extent ◯ Little extent ◯ Some extent ◯ Large extent ◯ Very large extent

Did you lack any specific information? ……………………………………………………………

……

What did you think of the technical design of the e-learning resource? ……………………………………………..

……

……

……

Do you have any suggestions for improvement to further develop of the e-learning resource?

……

……

……

Other comments: ………..

……

……

……

……

……

……

Would you recommend other students to carry out the e-learning resource in preparation to their first clinical placement ?

◯ yes ◯ no

Thank you for your participation!
